# Anatomical Pursuits

**Published:** 1853-10

**Authors:** 


*Anatomical Pursuits.* — The October number of the Knickerbocker magazine has an article on "How they make Doctors." The writer of this little sketch brings in a casual defense of anatomical study, and castigates the Mohawk dutchmen, anti-renters, *Van Schoonhovens*, &c., of whom he says there are always enough in the legislature, to defeat any bill for the relief of the profession. We suggest, that during the present winter, the profession should unite and demand from their representatives, such a law as will enable them to prosecute the study of anatomy in a legal manner. Were our county societies, at their winter meeting, to make this a matter of discussion, and should they unitedly demand the passage of a bill giving the bodies of those dying in prison and alms-houses, unclaimed by relatives, they would secure a successful issue. The medical profession has within its ranks influence and talent enough to carry such a measure without difficulty, were its strength put forth. And one of the strongest arguments for such a law, would be the fact that it would remove all excuse for those violations of the sanctity of the grave, which occasionally shock the sense of community, and give birth to unjust and injurious suspicions directed toward our whole fraternity. The profession, (and we speak not only for the colleges, but for every physician in the study,) has a right to this concession. Its public services are unequaled by any other occupation—its public rewards are nothing. We ask for no favoritism, for no endowments. The present law was conceived in an honest, but mistaken spirit, and honest but mistaken prejudices continue to operate. Probably violations of the present law are not very frequent. Evasions of its letter and spirit are more common. It is an unconscious materialism which maintains this penal statute; and we are confident that a well-directed and energetic appeal to the good sense and patriotism of an intelligent legislature, would bring about the change we ask. The profession is at present loaded down by laws antagonistic to its progress and its interests. Let us have some compensation for these in privileges granted, Messrs. Legislators, or the consequences will react upon community. The tyranny and oppression of a class always produce its degradation. Make us freemen in this respect, and a blessing will be conferred, not so much on the profession as on all suffering humanity which relies on its skill for relief.

